# Editorial: Nanomaterials for the diagnosis and therapy of viral infections

**DOI:** 10.3389/fbioe.2023.1192393

**Published:** 2023-04-03

**Authors:** Giuseppe Bardi, Donato Zipeto, Monica Neagu, Dragana Nikitovic

**Affiliations:** ^1^ Nanobiointeractions and Nanodiagnostics, Istituto Italiano di Tecnologia, Genova, Italy; ^2^ Department of Neurosciences, Biomedicine and Movement Sciences, University of Verona, Verona, Italy; ^3^ Doctoral School, Faculty of Biology, University of Bucharest, Bucharest, Romania; ^4^ Colentina Clinical Hospital, Bucharest, Romania; ^5^ “Victor Babes” National Institute of Pathology, Bucharest, Romania; ^6^ Medical School, University of Crete, Heraklion, Greece

**Keywords:** nanomaterials, virus, infection, diagnosis, therapy

Viral infections have been mournful protagonists of pandemics throughout history. The human immune system eventually adapted to the newly evolved viral strains, defeating diseases at high costs in terms of lives and productivity. Vaccines have been the best defense against viral infections; however, their development requires sufficient time for testing and validation. As we have sadly learned from the COVID-19 pandemic, several technologies can help to save lives and prepare us for future unexpected infections. In particular, nanotechnologies substantially contribute to the diagnosis and treatment of viral diseases. For example, nanomaterials have been widely used to detect SARS-CoV-2 infection and to deliver different types of vaccines, highly protecting humans from the risk of harmful diseases. This Research Topic collects research articles and reviews covering different aspects of nanotechnology applied to viral infection.

A comprehensive review article by Valenzuela-Fernandez et al. titled “Nanomaterials to combat SARS-CoV-2: Strategies to prevent, diagnose and treat COVID-19” describes the different fields of medicine in which applications of nanomaterials have helped to weaken the pandemic. The review presents the “pneumonia of unknown etiology” from a historical perspective of the discoveries and the technological approaches implemented during the global spreading. The paper highlights the importance of nanomaterial-based devices, from hospital identification of the virus to mask protection from infectious respiratory droplets. Moreover, the crucial role of nano-carriers for vaccine delivery and their tailored sustained immune system modulation are described.

In the late stage of the pandemic, point-of-care tests have been developed for inexperienced users to enable rapid self-diagnosis, speeding up decision processes and facilitating everyday life. These easy-to-use portable devices are often based on nanoparticles that increase their sensitivity. A specific review on nanoparticle-enhanced lateral-flow devices, titled “Rapid detection of SARS-CoV-2: The gradual boom of lateral flow immunoassay” by He et al., summarizes the current research progress on gold nanoparticle-based strips to detect SARS-CoV-2, describing advantages and limitations of different detection systems on the market. The authors also consider further improvements by combining CRISPR/Cas9 technology to achieve convenient and rapid assays with high sensitivity for the detection of future viruses.

Despite the importance of vaccines, a research article titled “Silver nanoparticles with excellent biocompatibility block pseudotyped SARS-CoV-2 in the presence of lung surfactant” by Gupta et al. shows the therapeutic properties of silver nanoparticles (AgNPs) to block SARS-CoV-2. AgNPs pre-incubated with pulmonary surfactants can prevent infection, as demonstrated in two different *in vitro* tests. In addition to the description of the neutralization mechanism by AgNP-mediated alteration of the Spike protein complex, the article provides a detailed characterization of the particles. AgNP stability and biocompatibility are key aspects of therapeutic applications. The role of the nanoparticle polymer coating and the protein corona under relevant media conditions were also carefully studied by the authors and represent important Research Topic for all future medical applications of nanomaterials. Metal-NPs always require careful analysis of their potential toxicity. A thorough characterization of nano-bio interactions allows exploiting the remarkable chemical characteristics of nanomaterials, reducing their toxicological risk within the safety threshold.

The Research Topic is further supported by a massive bibliometric analysis of nanomedicines applied to respiratory diseases, conducted by Yang et al. and titled “A scientometrics study of the nanomedicines assisted in respiratory diseases.” Trends in the literature show the future role of mRNA vaccines delivered by nano-carriers along with theranostic nanomaterial-based systems as crucial technologies for applications in respiratory disease treatments.

Nowadays, the relevance of nanomaterials in viral infection is rapidly growing, with many diagnostic and therapeutic products already on the market. The prevention of future viral pandemics could profoundly benefit from the interdisciplinary approaches of nano-bioscience research and their applications.

